# SNSVM: SqueezeNet-Guided SVM for Breast Cancer Diagnosis

**DOI:** 10.32604/cmc.2023.041191

**Published:** 2023-08-30

**Authors:** Jiaji Wang, Muhammad Attique Khan, Shuihua Wang, Yudong Zhang

**Affiliations:** 1 School of Computing and Mathematical Sciences, University of Leicester, Leicester, LE1 7RH, UK; 2 Department of Computer Science, HITEC University Taxila, Taxila, Pakistan; 3 Department of Information Systems, Faculty of Computing and Information Technology, King Abdulaziz University, Jeddah, 21589, Saudi Arabia

**Keywords:** SqueezeNet, support vector machine, breast cancer diagnosis

## Abstract

Breast cancer is a major public health concern that affects women worldwide. It is a leading cause of cancer-related deaths among women, and early detection is crucial for successful treatment. Unfortunately, breast cancer can often go undetected until it has reached advanced stages, making it more difficult to treat. Therefore, there is a pressing need for accurate and efficient diagnostic tools to detect breast cancer at an early stage. The proposed approach utilizes SqueezeNet with fire modules and complex bypass to extract informative features from mammography images. The extracted features are then utilized to train a support vector machine (SVM) for mammography image classification. The SqueezeNet-guided SVM model, known as SNSVM, achieved promising results, with an accuracy of 94.10% and a sensitivity of 94.30%. A 10-fold cross-validation was performed to ensure the robustness of the results, and the mean and standard deviation of various performance indicators were calculated across multiple runs. This model also outperforms state-of-the-art models in all performance indicators, indicating its superior performance. This demonstrates the effectiveness of the proposed approach for breast cancer diagnosis using mammography images. The superior performance of the proposed model across all indicators makes it a promising tool for early breast cancer diagnosis. This may have significant implications for reducing breast cancer mortality rates.

## 1 Introduction

Breast cancer [1–4] is a significant global health concern, affecting millions of women every year [5,6]. Timely identification of breast cancer is crucial for successful treatment. Traditional methods of diagnosis are time-consuming, expensive, and potentially invasive for the patient. The growing interest in machine learning techniques is spurring ongoing research into new and improved models for breast cancer diagnosis, which will lead to improvements in the accuracy and efficiency of medical diagnosis and a reduction in the need for invasive procedures [7].

Machine learning (ML) [8–10], particularly convolutional neural networks (CNNs) [11–13], has shown encouraging results in medical image analysis tasks. However, the high number of parameters in most used models may hinder their widespread use in clinical settings. SqueezeNet [14,15], a lightweight CNN architecture, is designed to achieve high accuracy in image classification while minimizing the number of parameters needed in the network. By reducing the number of parameters, SqueezeNet can improve the computational efficiency of the network and make it easier to deploy on devices with limited resources.

Support vector machine (SVM) [16,17], a commonly utilized ML technique for classification tasks, has achieved significant success in various applications [18–20]. SVM is useful in situations where the data is non-linearly separable, as it can employ kernel functions to map the input data into a higher-dimensional feature space where linear separation is possible. SVM has demonstrated strong capabilities in a range of medical image analysis tasks [21,22], particularly in the area of breast cancer diagnosis [23]. In the proposed model, the SVM classifier is used to classify the features extracted by SqueezeNet.

The use of ML methods for assisting in the diagnosis of breast cancer has become increasingly popular. This research [24] developed a novel approach for the automatic classification of thermal images using thermal-mammogram image processing techniques and smart devices. The proposed model outperforms other classifiers, achieving high precision, accuracy, and recall scores of 98.44%, 98.83%, and 100%, respectively, according to comparative analysis. This study [25] proposed a medical IoT-based system for early detection by hyperparameter-optimized neural networks. The system utilized both SVM and multilayer perceptron (MLP) to compare baseline classifiers for malignant and benign classification. The proposed method using CNN achieved a high accuracy of 98.5% in classifying. This study [26] proposed a deep learning cascaded feature selection framework to overcome the feature dimensionality curse problem. CNNs that were pre-trained were used to extract features from breast mammograms. The framework used a univariate strategy to select optimized key features with statistical significance, overcoming the curse of dimensionality and multicollinearity issues. The evaluation of the proposed framework yielded highly promising results, demonstrating the effectiveness of deep learning in breast cancer classification

Chen et al. [27] developed an automatic program using wavelet energy entropy and linear regression classifier that showed superior results in detecting breast abnormalities. Guo et al. [28] used wavelet energy to extract features, leading to improved accuracy in distinguishing benign and malignant tumors. Samala et al. [29] investigated the generalization error of a deep CNN that utilized transfer learning to classify mammogram images as benign or malignant. The proposed custom-designed CNN network [30] achieved impressive accuracy in distinguishing between metastatic and non-metastatic cells. The proposed framework by Baghdadi et al. [31] for breast cancer classification uses CNN and transfer learning. Eight classical pre-trained models were compared to arrive at the optimal choice. Manta ray foraging optimization achieved high accuracy on both histological and ultrasound data. The results of this study [32] showed that the sliced-Wasserstein autoencoder might be a promising approach for breast ultrasound image anomaly detection. However, further research is needed to address the challenge of reducing false positives in the reconstruction-based approach.

After reviewing these studies, it is evident that researchers have been consistently making advancements in utilizing deep learning for breast cancer classification. Nevertheless, the issue that the CNN model involves many parameters has remained. With increased parameters, the model becomes more complex, requiring more computational and storage resources for training and inference.

The proposed model overcomes the limitations of the previous methods. Compared to the deep learning methods mentioned earlier, the SVM approach guided by the lightweight SqueezeNet has fewer parameters. This is particularly important in medical image analysis, as computing resources always are limited. In all, this study has several novel contributions:

a) The proposed novel SNSVM can effectively diagnose breast cancer using mammography images and has the potential to help reduce breast cancer mortality rates.
b) Outperforming state-of-the-art models with higher accuracy and specificity while maintaining sensitivity.

## 2 Dataset and Preprocessing

Although not extensively used, the mini-MIAS dataset [33] is a valuable resource for classifying breast abnormalities. It encompasses 322 single-breast mammogram slices, each boasting a size of 1024 × 1024 pixels. Within the dataset, 113 images are labeled as abnormal, while the remaining 209 are normal. A selection of 100 images of healthy breasts and 100 images of diseased breasts was randomly made to form the input dataset to address the issue of the imbalanced dataset.

**Figure 1:**
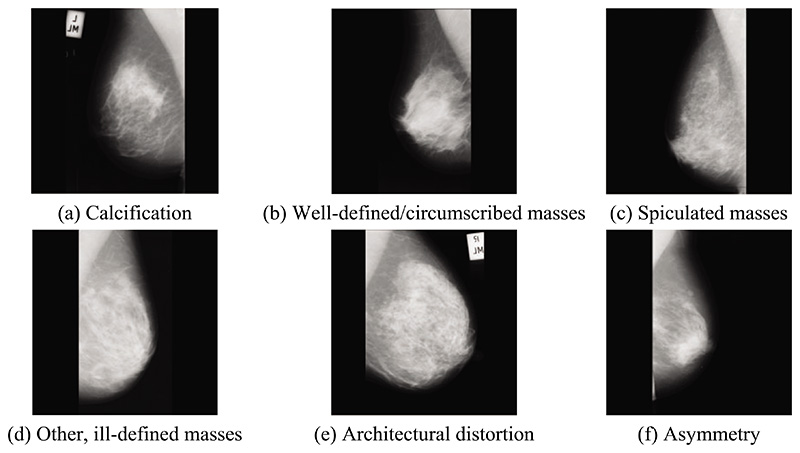
Examples of abnormal breast types in the mini-MIAS dataset

The dataset includes six categories of abnormal mammography images, exemplified in Fig. 1. In preprocessing, multiple data augmentation (MDA) is used [34,35]. MDA involves generating new images from existing data by applying various transformations to increase the size of a training dataset artificially. Although MDA does not directly improve the data’s quality, it provides a larger and more diverse image dataset for the model to learn from.

## 3 Methodology of SNSVM

### 3.1 Fire Module and SqueezeNet with Complex Bypass

SqueezeNet [14,36] is chosen as the base architecture for this project due to its small size and low number of parameters. Compared to larger models like AlexNet [37–39], VGG [40,41], or ResNet [42–44], SqueezeNet has a significantly smaller number of parameters, making it easier to train on limited computational resources. Table 1 compares the number of parameters of various deep learning models.

Additionally, SqueezeNet has achieved comparable accuracy to larger models on image classification tasks [45,46]. With these advantages, SqueezeNet is a suitable choice for this project.

**Figure 2:**
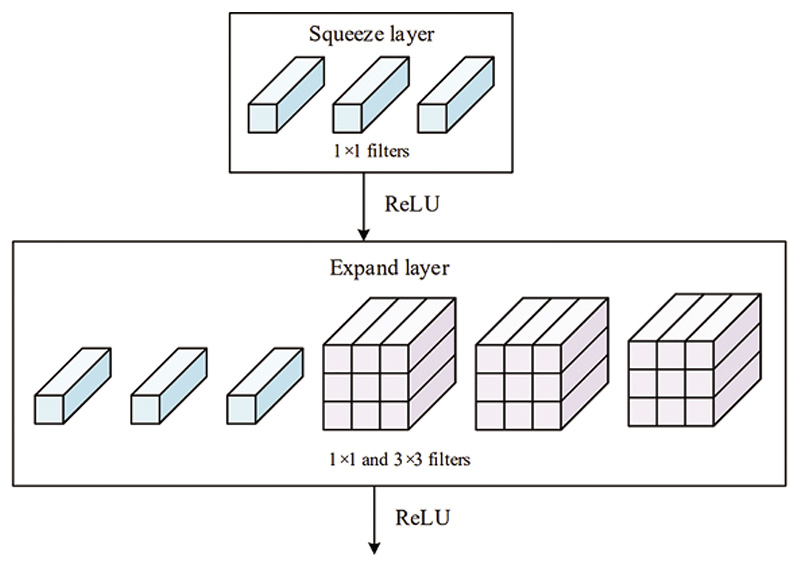
Structure of fire module

The fire module [47] is a fundamental building block in the SqueezeNet architecture, as shown in Fig. 2. The module contains two parts of convolutions, namely the squeeze layer and the expanding layer. The purpose of the 1 × 1 convolutional in the squeeze layer is to capture inter-channel relationships and is analogous to a fully connected layer operating on the channel dimension.

**Figure 3:**
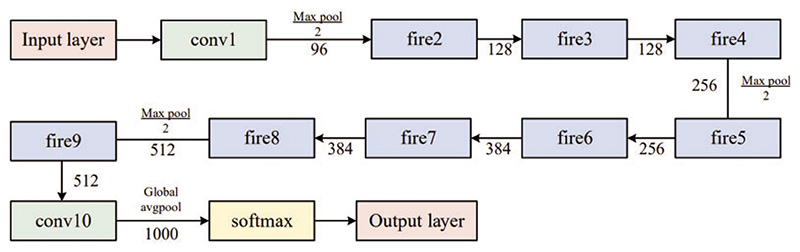
Original SqueezeNet structure

**Figure 4:**
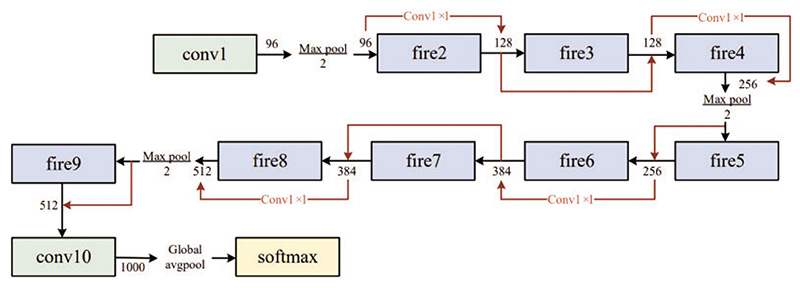
SqueezeNet with complex bypass

The expand layer aims to increase the number of channels. It combines 1 × 1 and 3 × 3 convolutions, allowing for a deeper representation of features. It is important to note that the expanding layer maintains the spatial dimensions of the input feature maps but increases the number of output channels. Figs. 3 and 4 compare the performance of the SqueezeNet with and without complex bypass.

The fire module has a limitation in cases where the number of input channels is identical to the number of output channels. This situation restricts the options for the fire module, which can only employ a straightforward bypass connection. If the number of input and output channels differs, a complex bypass connection must be used, which may result in a less efficient network and higher computational cost.

### 3.2 SqueezeNet-Guided SVM

In this study, SVM is trained on the SqueezeNet features extracted from the mini-MIAS dataset and is used to classify the mammogram images into six categories. SVM is a widely used binary classifier that is known for its high accuracy in both linear and non-linear classification tasks, but it can also be used for multi-class classification.

**Figure 5:**
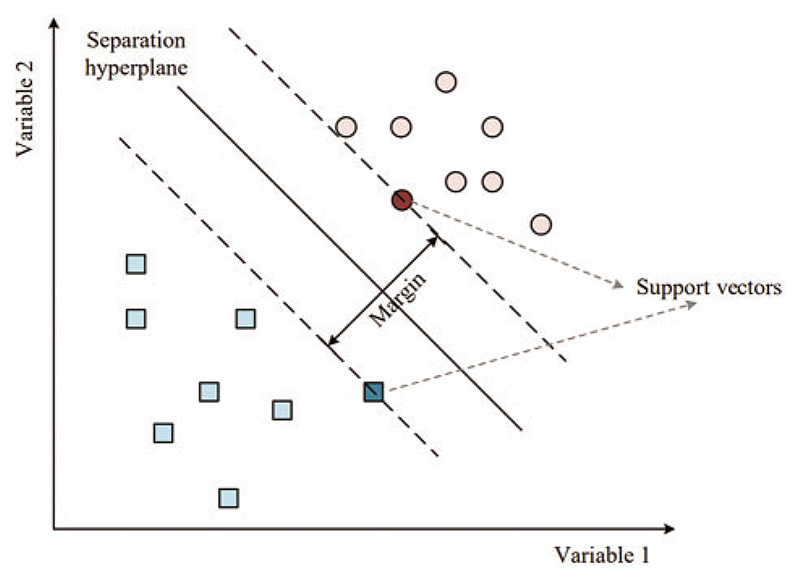
Separation hyperplane of SVM

The primary concept behind SVM is to construct a hyperplane or a set of hyperplanes that can effectively separate different classes in the input space, as shown in Fig. 5.

The equation for the linear decision hyperplane is:

(1) $$f\;(x)=w^{T}x+b,$$

where the weight vector is denoted by *w*, the bias is denoted by *b*, *w^T^* denotes the transpose of the weight vector, *x* represents the input vector that contains the features of a data point.

In classification tasks, SVM seeks to identify the optimal hyperplane for separating data points into distinct classes, with the widest possible margin between the closest data points of each class. The margin is defined as the maximum distance between the hyperplane and the closest data points in the training set, and it is an important concept in SVM [48]. A larger margin indicates that the classifier is more robust to new data points. o find the optimal hyperplane, SVM seeks to minimize the classification error while maximizing the margin, which can be achieved by finding the optimal values of *w* and *b*.

The optimization problem of SVM can be formulated as follows:

(2) $$\{\begin{array}{l} \min_{w,b}\frac{1}{2}w^{T}w+C\;\sum_{i=1}^{n}\zeta_{i}\\ \text{subject}\;\text{to}\;y_{i}\left(w^{T}x_{i}+b\right)\geq 1-\zeta_{i} \end{array},$$

where the input vector is denoted by *x_i_*, while *y_i_* represents the corresponding output label. The slack variable *ζ_i_* is also used in SVMs, and its role is to allow some training examples to be misclassified within certain limits. The hyperparameter *C* is introduced to balance between maximizing the margin and minimizing the classification error. It determines the amount of penalty to be imposed for misclassification. Finally, *n* refers to the total number of training samples. The first term in the objective function $\frac{1}{2}w^{T}w$ encourages the margin to be maximized, while the second term $C\;\sum_{i=1}^{n}\zeta_{i}$ penalizes the misclassification of training examples. The Lagrangian for this problem can be written as:

(3) $$L\left(w,b,\zeta ,\alpha ,\beta \right)=\frac{1}{2}w^{T}w+C\;\sum_{i=1}^{n}\zeta_{i}-\sum_{i=1}^{n}\alpha_{i}\left[y_{i}\left(w^{T}x_{i}+b\right)-1+\zeta_{i}\right]-\sum_{i=1}^{n}\beta_{i}\zeta_{i},$$

where *α* = (*α*_1_, …, *α_n_*) and *β* = (*β*_1_, …, *β_n_*) are non-negative Lagrange multipliers. By setting the partial derivatives of *L* with respect to *w*, *b*, and *ζ* and setting them to zero, get:

(4) $$w=\sum_{i=1}^{n}\alpha_{i}y_{i}x_{i}$$

Considering the Karush-Kuhn-Tucker condition [49], the dual Lagrangian can be derived as follows:

(5) $$\{\begin{array}{l} \max_{\alpha }\;L\left(\alpha \right)=\sum_{i=1}^{n}\alpha_{i}-\frac{1}{2}\sum_{i=1}^{n}\sum_{j=1}^{n}\alpha_{i}\alpha_{j}y_{i}y_{j}{x_{i}}^{T}x_{j}\\ \text{subject}\;\text{to}\;\sum_{i=1}^{n}\alpha_{i}y_{i}=0 \end{array}$$

In the case of non-linear SVM, kernel functions are introduced to map the input data into a higher-dimensional space. The optimization problem, in this case, becomes:

(6) $$\{\begin{array}{l} \max_{\alpha }\;L\left(\alpha \right)=\sum_{i=1}^{n}\alpha_{i}-\frac{1}{2}\sum_{i=1}^{n}\sum_{j=1}^{n}\alpha_{i}\alpha_{j}y_{i}y_{j}G\left(x_{i},x_{j}\right)\\ \text{subject}\;\text{to}\;\sum_{i=1}^{n}\alpha_{i}y_{i}=0,0<\alpha_{i}<C \end{array},$$

where *G* (*x_i_*, *x_j_*) is the kernel function. The most used kernel functions are:

Gaussian kernel:

(7) $$G_{Gau}\left(x_{i},x_{j}\right)=\exp\left(-\frac{{\left\|x_{i}-x_{j}\right\|}^{2}}{2\sigma^{2}}\right),$$

where *σ* is a hyperparameter that controls the smoothness of the decision boundary.

Polynomial kernel:

(8) $$G_{Pol}\left(x_{i},x_{j}\right)={\left({x_{i}}^{T}x_{j}+h\right)}^{d},$$

where *d* is the degree of the polynomial. *h* refers to a free parameter that controls the degree of the polynomial expansion.

### 3.3 Cross-Validation

Cross-validation is widely used in machine learning and statistics to estimate the performance of a predictive model on a limited dataset. The basic idea is to divide the dataset into two or more folds, one for testing the model and the other for training it [50].

K-fold cross-validation is a commonly used method of cross-validation. The dataset *X* is split into *K* almost equally sized folds *D* = {*D*_1_, *D*_2_, …, *D_K_*}. The model is then trained on *K* − 1 folds, then tested on the remaining fold [51]. This process is repeated *K* times, each time using a different fold as the test set.

In the *k*-th trial, the training set *X*_*k*_*tr*_ = {*D*_1_, *D*_2_, …, *D*_*k*−1_, *D*_*k*+1_, …, *D_K_*} contains all the samples from *X* except for the samples in the *k*-th fold. The test set *X*_*k*_*te*_ = {*D_k_*} contains the samples from the *k*-th fold.

In the end, the model’s performance is assessed by taking the average of the accuracy values obtained over *K* times [52]:

(9) $$m=\frac{1}{K}\sum_{g=1}^{K}M_{g},$$

where *M_g_* represents the test indicator obtained on the *g*-th fold.

**Figure 6:**
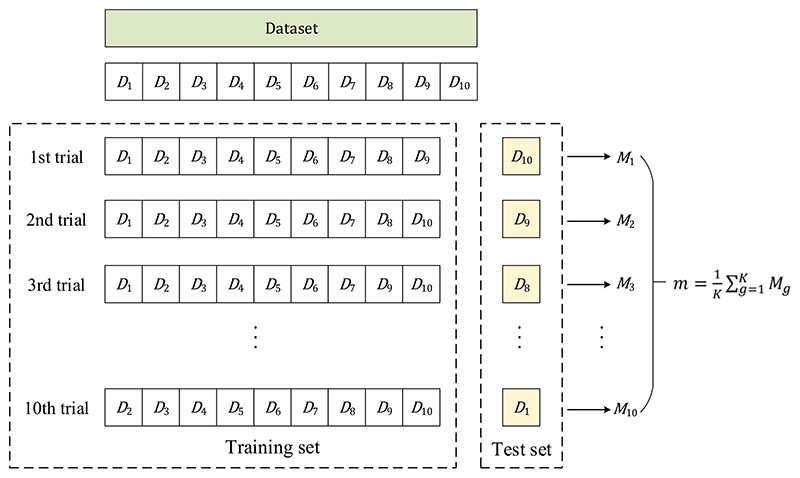
Illustration of 10-fold cross-validation

K-fold cross-validation is a useful approach for assessing the generalization performance of a model and tuning the hyperparameters of an algorithm or model. It is a powerful technique for evaluating models, especially when the dataset is small. It helps to reduce overfitting by using multiple validation sets. Fig. 6 shows the illustration of K-fold cross-validation (*K* = 10). Table 2 lists the symbols and their corresponding meanings.

## 4 Experiments and Results

### 4.1 Results of MDA

**Figure 7:**
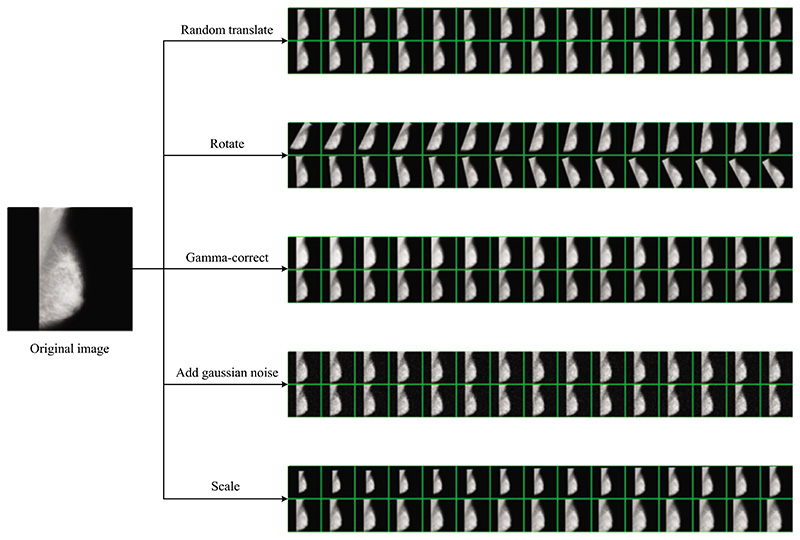
Results of MDA

The results of the MDA can be observed in Fig. 7, which includes five data augmentation methods: random translation, rotation, Gamma-correction, and Gaussian noise injection, scaling. These methods have increased the diversity of the dataset, addressing the limitation of having a small dataset size.

Random translation allows the objects or patterns in the images to shift within the image space, enabling the model to learn to recognize objects regardless of their specific location. Rotation augmentation introduces variations in the orientation of objects. Gamma correction can adjust the image brightness, which can be useful for handling varying lighting conditions. Gaussian noise injection adds random noise to the images, simulating noise commonly present in real-world data. Scaling augmentation alters the size of objects in the images, allowing the model to learn to recognize objects at different scales.

### 4.2 10-Fold Cross-Validation

The SNSVM model is evaluated by running 10-fold cross-validation ten times. The results are listed in Table 3. The mean and standard deviation (MSD) of all runs are calculated to display the performance of the model and the fluctuations from multiple runs.

The sensitivity (Sen) is 94.30% ± 1.34%, the specificity (Spc) is 93.90% ± 1.79%, the precision (Prc) is 93.95% ± 1.65%, the accuracy (Acc) is 94.10% ± 1.13%, the F1 score is 94.11% ± 1.11%, the MCC is 88.22% ± 2.23%, and the FMI is 94.12% ± 1.10%.

Results rounded to two decimal places strikes a balance between result accuracy and readability. Overly precise results make data difficult to understand. The indicators presented in Table 3 suggest that the proposed model demonstrates good performance. After multiple runs, the final indicators obtained by averaging various indicators are consistently high, exceeding 90, except MCC.

The important indicator, accuracy, has an average value of 94.10% across multiple runs. The small standard deviation values indicate that there is little fluctuation in the same indicator across multiple runs. MCC is currently showing the largest fluctuation, only 2.23%. These MSD results for multiple indicators indicate that the SNSVM model is effective in the task.

### 4.3 Convergence Plot

**Figure 8:**
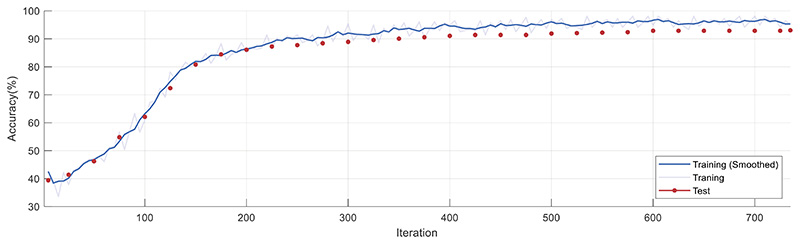
Accuracy convergence plot with iteration number

The accuracy curves in Fig. 8 are obtained by iterating over the experiment process and plotted using Matlab. The light blue indicates the actual training results. The smoothed training results are indicated by dark blue.

The red dots indicate the test accuracy for each iteration. Fig. 8 shows that the accuracy increases rapidly during the initial 0–200th iteration. In the 200th–400th iteration, the curve improves at a slower rate. After the 400th iteration, the test and training results remained consistently above 90%.

### 4.4 Receiver Operating Characteristic (ROC) Curve

**Figure 9:**
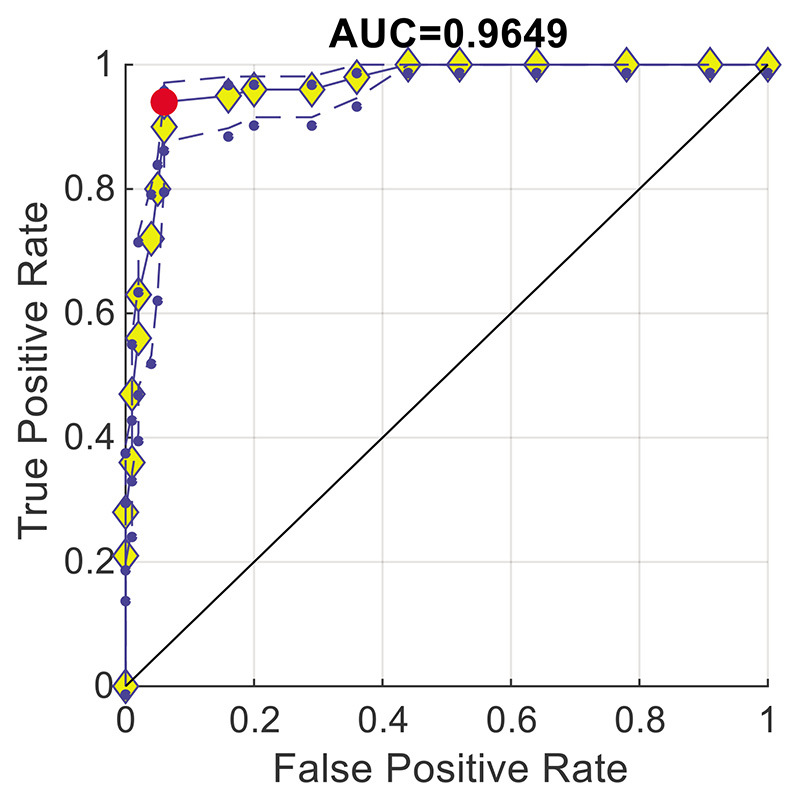
ROC curve

The ROC curve is a visual tool for evaluating the performance of a binary classification model. It illustrates the trade-off between the true positive rate (TPR) and the false positive rate (FPR). The ROC curves in Fig. 9 are plotted by combining TPR and FPR from the ten runs. TPR is calculated as the number of true positive predictions divided by the total number of actual positives. FPR stands for False Positive Rate and is calculated as the number of false positive predictions divided by the total number of actual negatives. A high TPR indicates that the model is correctly identifying positive cases, while a low FPR indicates that the model is not falsely identifying negative cases as positive.

The area under the ROC curve (AUC) is a common metric used to summarize the performance of a classifier. An AUC value of 1 indicates perfect discrimination, which is practically impossible. An AUC value below 0.5 means worse performance than random guessing. Consequently, the AUC value of a model’s ROC curve increases as the curve approaches the upper left corner (0, 1), indicating a better classification performance of the model.

To better visualize whether the AUC is greater than 0.5, a solid black line connecting (0, 0) and (1, 1) is added. The area below this black line is 0.5. Obviously, the ROC curve obtained using SNSVM has an AUC = 0.9649 greater than 0.5. The cut-off point is used to determine the optimal classification threshold for the model, which balances the trade-off between TPR and FPR. The most prominent red point in Fig. 9 represents the cut-off point of the ten runs.

### 4.5 Comparison with State-of-the-Art Models

**Figure 10:**
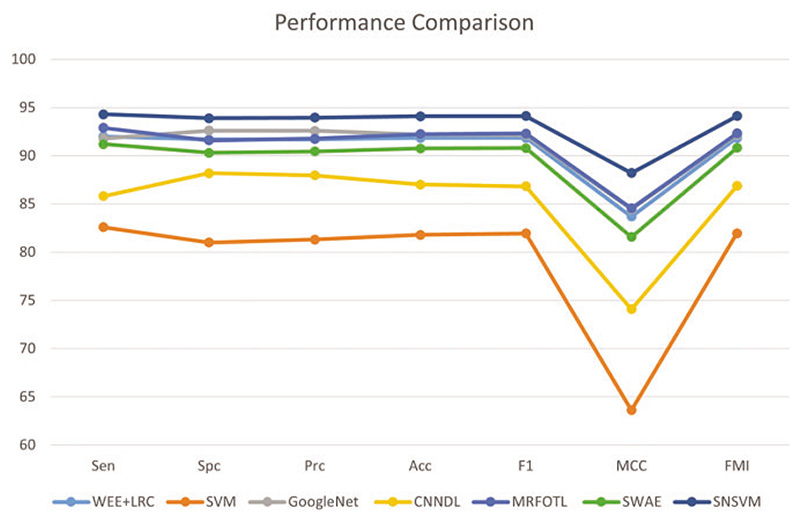
Comparison of SNSVM with other models

SNSVM is compared with state-of-the-art models, which include WEE + LRC [27], SVM [28], GoogleNet [29], CNNDL [30], MRFOTL [31], and SWAE [32], as shown in Table 4. A comparison of the results reveals that in each of the indicators, the proposed SNSVM gets the best results, but there is some fluctuation in the multiple validations. Based on Table 4, Fig. 10 is generated.

## 5 Conclusions

A model is proposed in this study, utilizing an SVM classifier guided by SqueezeNet with complex bypass. The dataset used for training is the publicly available breast cancer image dataset, mini-MIAS. Evaluation indicators include Sen, Spc, Prc, Acc, F1, MCC, and FMI. The experimental findings demonstrate that the proposed model surpasses the existing state-of-the-art models in all the performance metrics and exhibits reasonable fluctuations.

To enhance the performance of the SNSVM model, exploration of different algorithms and improvement of existing ones are planned to capture underlying patterns in the data more effectively. Thorough feature engineering will be conducted to extract relevant information and reduce noise. Furthermore, fine-tuning the model’s hyperparameters will be pursued to achieve improved results. The aim is to optimize the model and increase its predictive power through these steps, resulting in a more effective decision.

## Figures and Tables

**Table 1:** Comparison of parameter counts for popular CNN model

Model	Number of parameters
AlexNet	61 million
VGG-16	138 million
Inception-v3	23 million
ResNet-50	25 million
MobileNet	4.2 million
SqueezeNet	1.2 million

**Table 2:** Symbols and their corresponding meaning

Symbol	Meaning
*w*	The weight vector
*b*	The bias
*x*	The input vector that contains the features of a data point
*i*	An index that iterates over all the data points in the dataset
*x_i_*	The *i*-th input vector
*y_i_*	The output label corresponding to the *i*-th input vector
*ζ_i_*	A non-negative slack variable associated with the *i*-th training example
*C*	A hyperparameter that controls the trade-off between maximizing the margin and minimizing the classification error
*α*	Non-negative Lagrange multiplier
*β*	Non-negative Lagrange multiplier
*n*	The number of training samples
*w^T^*	The transpose of the weight vector
*σ*	A hyperparameter that controls the smoothness of the decision boundary
*d*	The degree of the polynomial
*h*	A free parameter trading off the influence of higher-order *vs*. lower-order terms in the polynomial
*X*	The entire input set
*K*	The number of folds in K-fold cross-validation
*D*	The set of *K* almost equally sized folds into which the dataset *X* is divided
*M_g_*	The test indicator obtained on the *g*th fold
*X_k_te_*	The test set at *k*-th trial
*X_k_tr_*	The training set at *k*-th trial
*m*	The fold-average test indicator
*g*	The index that ranges from 1 to *K*

**Table 3:** Results of 10 × 10-fold cross-validation (Unit: %)

Run	Sen	Spc	Prc	Acc	F1	MCC	FMI
1	94.00	93.00	93.07	93.50	93.53	87.00	93.53
2	93.00	95.00	94.90	94.00	93.94	88.02	93.94
3	95.00	94.00	94.06	94.50	94.53	89.00	94.53
4	95.00	95.00	95.00	95.00	95.00	90.00	95.00
5	93.00	94.00	93.94	93.50	93.47	87.00	93.47
6	96.00	93.00	93.20	94.50	94.58	89.04	94.59
7	94.00	94.00	94.00	94.00	94.00	88.00	94.00
8	92.00	94.00	93.88	93.00	92.93	86.02	92.93
9	96.00	97.00	96.97	96.50	96.48	93.00	96.48
10	95.00	90.00	90.48	92.50	92.68	85.11	92.71
MSD	94.30 ± 1.34	93.90 ± 1.79	93.95 ± 1.65	94.10 ± 1.13	94.11 ± 1.11	88.22 ± 2.23	94.12 ± 1.10

**Table 4:** Comparison of the SNSVM with other state-of-the-art models (Unit: %)

Model	Sen	Spc	Prc	Acc	F1	MCC	FMI
WEE + LRC [27]	92.00	91.70	91.72	91.85	91.86	83.70	91.86
SVM [28]	82.60	81.00	81.30	81.80	81.94	63.61	81.95
GoogleNet [29]	91.80 ± 1.99	92.60 ± 1.65	92.58 ± 1.49	92.20 ± 0.79	92.16 ± 0.83	84.44 ± 1.57	92.18 ± 0.82
CNNDL [30]	85.80 ± 2.62	88.20 ± 2.15	87.97 ± 1.82	87.00 ± 1.13	86.83 ± 1.23	74.08 ± 2.25	86.86 ± 1.22
MRFOTL [31]	92.90 ± 1.66	91.60 ± 2.41	91.77 ± 2.07	92.25 ± 1.03	92.30 ± 0.97	84.55 ± 2.01	92.32 ± 0.96
SWAE [32]	91.20 ± 2.10	90.30 ± 2.36	90.45 ± 1.99	90.75 ± 0.82	90.79 ± 0.82	81.57 ± 1.64	90.81 ± 0.82
SNSVM (Ours)	94.30 ± 1.34	93.90 ± 1.79	93.95 ± 1.65	94.10 ± 1.13	94.11 ± 1.11	88.22 ± 2.23	94.12 ± 1.10
